# A Novel Method HPLC-DAD Analysis of the Contentsof Moutan Cortexand Paeoniae Radix Alba with Similar Constituents-Monoterpene Glycosides in Guizhi Fuling Wan

**DOI:** 10.3390/molecules191117957

**Published:** 2014-11-04

**Authors:** Shuyun Wang, Jian Huang, Huijuan Mao, Yuliang Wang, Rena Kasimu, Wei Xiao, Jinhui Wang

**Affiliations:** 1 School of Traditional Chinese Materia Medica & Key Laboratory of Structure-Based Drug Design and Discovery of Ministry of Education, Shenyang Pharmaceutical University, Shenyang 110016, China; E-Mails: wangshuyun0426@163.com (S.W.); maohuijuan123@163.com (H.M.); wangyuliang123@163.com (Y.W.); 2 School of Pharmacy, Xinjiang Medical University, Urumqi 830054, China; E-Mail: renakasimu@vip.sina.com; 3 Jiangsu Kanion Pharmaceut Co Ltd, Lianyungang 222001, China; E-Mail: xmb@kanion.com

**Keywords:** Moutan cortex, Paeoniae radix alba, GuizhiFuling Wan, monoterpeneglycosides, HPLC-DAD

## Abstract

A variety of traditional Chinese medical formulations contain two or more herbs from the same genus or family. Although these herbs may have a similar appearance and constituents, they usually have different pharmacodynamic actions. A series of qualitative and quantitative analysis methods are developed to determine one or more compounds for quality control of medicine. As far as we know, no method has been found to determine the real ratio of the two herbs along with the prescription. In this study, we used HPLC-DAD as a way to determine the content of Moutan cortex (**M**) and Paeoniae radix alba (**P**) in GuizhiFuling Wan (GZFLW). An effective, accurate and reliable HPLC-DAD method was developed for detecting the content of **M** and **P** in GZFLW through the analysis of four monoterpeneglycosides, namely, galloylpaeoniflorin (**1**), paeoniflorin (**2**), mudanpioside C (**3**) and benzoylpaeoniflorin (**4**). Due to the different UV characteristics of the compounds, the detection wavelength was 270 nm for **1** and **2**, while **3** and **4** were monitored at 254 nm and 230 nm, respectively. Four equations were put forward to describe the relationship between content of **M** as well as **P** and the four monoterpene glycosides in GZFLW. After validation, all the accuracies of the **M** and **P** contents in GZFLW were within 10%. The result showed that the method could be successfully applied to analyze the contents of **M** and **P** in GZFLW. Moreover, our method may be more widely used to control the quality of proprietary Chinese medicines, especially for those containing the same genus or family herbs, in industrial GMP production.

## 1. Introduction

Traditional Chinese medicines, playing an irreplaceable role in the health of people, have attracted more and more attention around the world. Some traditional Chinese medical formulations contain two herbs from the same family, such as Moutan cortex (**M**) and Paeoniae radix alba (**P**) in GuizhiFuling Wan (GZFLW), Asparagi radix and Ophiopogonis in Erdong Gao, Mastic and Myrrha in Qili Capsule, as well as Citrireticulataepericarpium and Aurantiifructusimmaturs in Ermuningke Wan [1]. Although the two herbs may have similar appearance and constituents, they may differ in their pharmacodynamic action. Within our knowledge, almost all the quality of traditional Chinese medical formulations is controlled through major or active constituents [2,3,4,5]. The methods for detecting quality are sensitive and available when the formulation has no common components in any of the constituent herbs, but how to determine the real ratio of the two herbs along with the prescription remains a difficult question. Therefore, effective and comprehensive analytical methods have to be developed, therefore controlling the ratio of the each herb to agree with the prescription. In this paper, an available and accurate method, high performance liquid chromatography method with photodiode array detector (HPLC-DAD), was applied for detecting the contents of **M** and **P** in GZFLW.

GZFLW was first described in Jin Kui Yao Lue written by theeminent Chinese physician Zhang Zhongjing during the Han Dynasty (220 A.D.). In pharmacological research, GZFLW has been reported to protect against brain ischemia-reperfusion injuries and NO-mediated neuronal death [6,7], to improve blood circulation and arteriosclerosis [8], regulate immunology in endometriosis [9], and inhibit cervical cancer [10]. Clinically, GZFLW has been applied to treat gynecological diseases, such as uterine fibroids, endometriosis, pelvic inflammatory disease, ovarian cysts, dysmenorrheal, and “oketsu” syndrome, the so-called blood stasis syndrome in postmenopausal women [11,12].

GZFLW is composed of five herbs (1:1:1:1:1, g/g), namely **M**, **P**, Cinnamomi ramulus, Poria and Persicae semen. **M** and **P**, both from the Paeoniaceae family and the *Paeonia* genus, contain similar constituents including galloylpaeoniflorin, paeoniflorin and benzoylpaeoniflorin [13,14,15], but have different pharmacodynamic actions, whereby galloylpaeoniflorin has apronounced radical scavenging effect [16] and inhibits phenylhydroquinone-induced oxidative DNA cleavage [17], but paeoniflorin could attenuate Aβ25-35-induced neurotoxicity in PC12 cells [18], and inhibit tumor invasion and metastasis in human hepatocellular carcinoma cells [19]. Mudanpioside C can be used for treating and preventing cardiovascular diseases [20].while benzoylpaeoniflorin inhibited the replication of hepatitis B Virus [21] and has lipoxygenase inhibitory and antioxidant activities [22].

**Figure 1:**
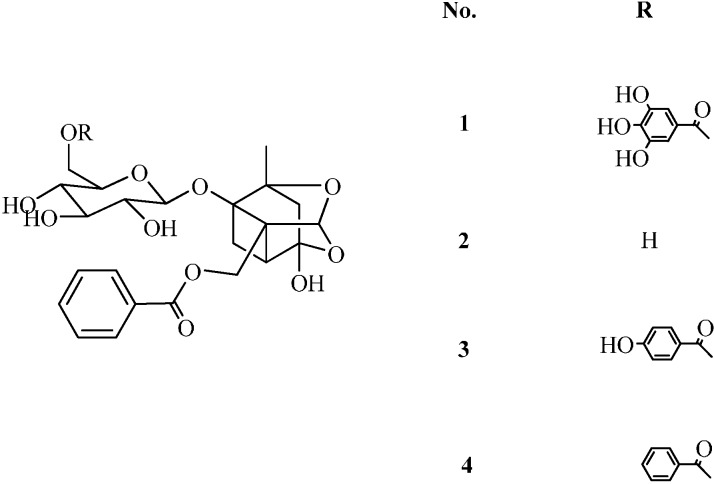
Chemical structures of galloylpaeoniflorin (**1**), paeoniflorin (**2**), mudanpioside C (**3**) and benzoylpaeoniflorin (**4**).

As to the quality control of GZFLW [23,24,25], several analytical methods have been only used to analyze the total contents of the common constituents from the two herbs, but not the ratio of the two herbs in the prescription. However, the contents of **M** and **P** in the prescription may exert a large influence on the clinical effect. Therefore, it is essential to determine the contents of **M** and **P** in GZFLW through analysis of four common monoterpene glycosides by HPLC-DAD. The structures of the four common monoterpene glycosides are shown in Figure 1. As we know, traditional Chinese medicine contents always vary according to the geographical origins, cultivation and harvesting methods, as well as post-harvesting processes, thereby the original medicinal materials of the proprietary Chinese medicine should be determined according to the analytical method.

## 2. Results and Discussion

### 2.1. Optimization of Sample Preparation

To determine the best extraction method for the four monoterpene glycosides with high recovery and no interference at the retention time, various solvents (methanol, acetone and ethyl acetate), methods (ultrasonic extraction and Soxhlet extraction) and times of Soxhlet extraction (1, 2, 3 and 4 h) were applied. As a result, the Soxhlet extraction with methanol for 3 h was selected as the optimum method. The results are shown in Table 1.

**Table 1:** Optimization of extraction method of GZFLW.

Extraction Method	Extractant	Time	Compounds (mg/g)				Total
		(h)	1	2	3	4	(mg/g)
Ultrasonic	Ethyl acetate	0.5	0.2581	4.5582	0.0805	0.7246	5.6213
Ultrasonic	Acetone	0.5	0.1656	2.1447	0.0394	0.6233	2.9730
Ultrasonic	Methanol	0.5	0.1371	2.5489	0.0985	0.7065	3.4910
Soxhlet	Methanol	1.0	0.4378	5.7462	0.0980	0.7399	7.0220
Soxhlet	Methanol	2.0	0.4593	5.6639	0.1016	0.7532	6.9780
Soxhlet	Methanol	3.0	0.5075	7.0481	0.1028	0.8105	8.4689
Soxhlet	Methanol	4.0	0.4810	6.6659	0.1000	0.7785	8.0254

### 2.2. Optimization of Chromatography Conditions

**Figure 2:**
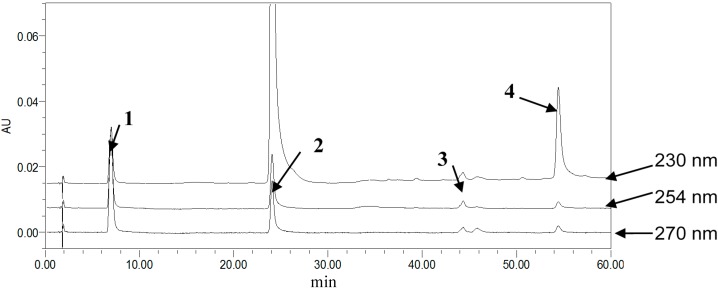
Stacked view of different detector wavelength HPLC chromatograms of mixed reference standards (from topto bottom: 230, 254, 270 nm). Column: SunFire^TM^ C18 (4.6 mm × 150 mm, 5 μm), temperature of 30 °C.

**Figure 3:**
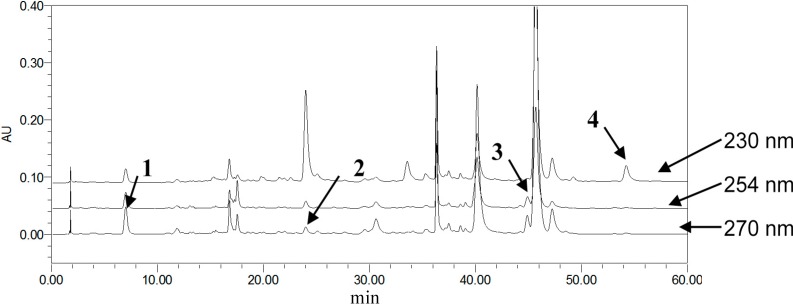
Stacked view of different detector wavelength HPLC chromatograms of GZFLW (from top to bottom: 230, 254, 270 nm).

**Figure 4:**
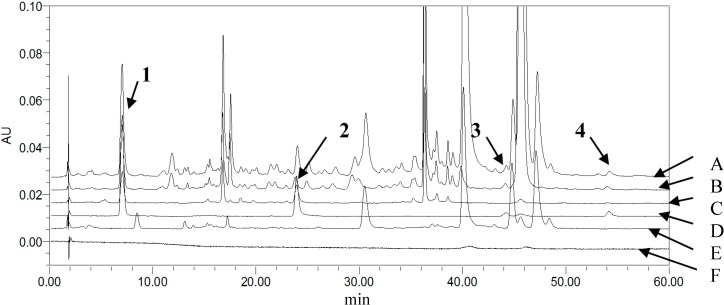
Stacked view of HPLC chromatograms (wavelength: 270 nm) of (A) GZFLW; (B) Moutan cortex; (C) Paeoniae radix alba; (D) mixed reference standards; (E) negative control; (F) Blank solvent (from up to down).

To achieve symmetric peak shapes and short run times for the simultaneous analysis of the four compounds, chromatographic conditions were optimized through different trials. In this respect, the column choice had a great influence on the compound separation, which was essential for the success of the method. During the development of methods, three reversed-phase columns, SunFire^TM^ C18 (5 μm, 4.6 × 150 mm), Agela Technologies Lnc. C18 (5 μm, 4.6 × 200 mm) and Kromasil ODSI C18 (5 μm, 4.6 × 250 mm), were tested with different mobile phase compositions. UV-Visible detection was applied over the wavelength range of 200–600 nm. Consequently, the selected wavelengths were 270 nm for **1** and **2**, 254 nm for **3** and 230 nm for **4**, and the SunFire^TM^ column with a gradient elution, methanol, and 0.3% phosphoric acid as the mobile phase, thereby providing the best balance of peak shape, sensitivity and retention time for each monoterpene glycoside. Examples of typical chromatograms wreshown in Figure 2, Figure 3 and Figure 4.

### 2.3. Simultaneous Determination of Multiponents: **1**, **2**, **3**, **4** in **M**, **P** and GZFLW

#### 2.3.1. Specificity

The specificity of the method was tested by comparing the chromatograms of a blank solution, the mixed working standard solution, the control solution, and the sample solution.

#### 2.3.2. Linearity, Limit of Detection (LOD) and Limit of Quantitation (LOQ)

Linearity was established by the injection of 2, 6, 10, 14, 18 and 22 μL of the mixed working standard solutions. It was also assessed by analyzing calibration curves with the least square linear regression of the integrated peak area (Y) *versus* monoterpeneglycoside content (X). All obtained correlation coefficients were above 0.999. The limits of detection (LOD) and quantification (LOQ) of each monoterpeneglycoside were determined at a signal-to-noise ratio (S/N) of 3 and 10, respectively. Detailed information regarding calibration curves, linear ranges, LOD and LOQ is presented in Table 2.

**Table 2:** Linearity, LOD, LOQ of tested compounds determined by the current method.

Compounds	Calibration Curves	*r*	Linear Range (ng)	LOD (ng)	LOQ (ng)
**1**	Y = 2.77 × 10^3^ X + 6.18 × 10^3^	0.9997	0.04–0.49	0.88	2.21
**2**	Y = 1.02 × 10^2^ X − 7.74 × 10^3^	0.9996	0.67–7.32	0.67	166.38
**3**	Y = 7.65 × 10^2^ X + 4.35 × 10^3^	0.9997	0.01–0.13	2.98	5.95
**4**	Y = 1.58 × 10^3^ X + 1.65 × 10^4^	1.0000	0.09–0.10	0.91	4.56

#### 2.3.3. Precision, Stability, Reproducibility and Recovery

The precision was evaluated by injecting 10 μL of the mixed working reference solution in six replicates in one day. Stability was tested with a GZFLW sample solution over 24 h (the time points of the injections were at 0, 2, 4, 6, 8, 12, and 24 h). The variations were reported as relative standard deviations (RSD in %). The reproducibility of the method was assessed within six independently prepared sample solutions and evaluated by the RSD value of each monoterpeneglycoside content in GZFLW. Recovery was tested in a set of six replicates by spiking the appropriate stock standard solutions into untreated GZFLW at the same concentration. As shown in Table 3, validation studies of the method proved that it had good precision and reproducibility, with RSD ranging from 1.13% to 1.69%, and 0.89% to 2.31%, respectively. It was also found that the four monoterpene glycosides in the GZFLW sample solution were all stable for 24 hours with a RSD of 1.20%–1.84%. The results of the recovery test indicated that the recoveries of the four monoterpeneglycosides were satisfactory, between 97.9% and 103.4% with RSD of 0.77%–2.42%.

**Table 3:** Precision, stability, recovery and reproducibility of the assay method.

Compounds	Precision		Stability	Recovery		Reproducibility	
	Concentrations (μg/mL)	RSD (%)	RSD (%)	Average (%)	RSD (%)	Average Content (mg/g)	RSD (%)
**1**	22.05	1.13	1.20	101.71	1.67	0.4972	1.30
**2**	332.80	1.53	1.47	99.93	1.94	6.9337	1.88
**3**	5.95	1.69	1.83	98.18	2.51	0.0782	2.37
**4**	45.60	1.21	1.24	100.39	1.00	0.7879	1.02

RSD refers to relative standard deviation.

#### 2.3.4. Quantification Analysis **1**, **2**, **3** and 4 in **M**, **P** and GZFLW

The contents of **1**, **2**, **3** and **4** in **M**, **P** and GZFLW with different weight ratios of **M** and **P** were quantified by the simultaneous determination of multiponents. The contents of each monoterpeneglycoside in **M**, **P** and GZFLW with different ratios of **M** and **P** were shown in Table 4. These results could be used to calculate the weight of **M** and **P** in GZFLW.

**Table 4:** Contents of **1**, **2**, **3**, **4** in **M**, **P** and GZFLW (mg/g).

Analysis	M	P	GZFLW with Different M/P Ratios										
Compounds			NO.1	NO.2	NO.3	NO.4	NO.5	NO.6	NO.7	NO.8	NO.9	NO.10	NO.11
**1**	1.6896	0.8322	0.4242	0.4235	0.4421	0.4654	0.4907	0.5116	0.5121	0.5466	0.5604	0.5807	0.5908
**2**	8.3567	15.4794	5.7133	5.4802	5.1281	5.3575	4.8711	4.9124	4.6395	4.4391	4.3825	4.0811	4.0044
**3**	0.3825	ND	0.0366	0.0444	0.0466	0.0622	0.0636	0.0806	0.0855	0.1031	0.1038	0.1066	0.1083
**4**	2.9510	0.3838	0.4417	0.4501	0.5083	0.5640	0.5806	0.6326	0.6996	0.8003	0.8697	0.8740	0.8983

ND (not detected).

### 2.4. Method Application and Validation: Analysis of the Weight of **M** and **P** in GZFLW

The contents of **M** and **P** in GZFLW were calculated according to following equations:

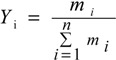
(1)

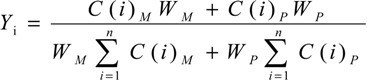
(2)

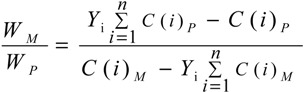
(3)

*C* (*i*) _*M*_ × *W*_*M*_ + *C* (*i*) _*P*_ × *W*_*P*_ = *m*_*i*_ (4)

where *I* was the number of the monoterpene glycoside, also known as **1**, **2**, **3** and **4**. *m_i_* (mg) was the weight of each monoterpene glycoside in GZFLW with the corresponding ratio of M and P. *Y_i_* was each monoterpene glycoside after accounting for the ratio of four monoterpene glycosides. *C(i)_M_* and *C(i)_P_* were the content of each monoterpene glycoside (mg/g) in **M** and **P**, while *W_M_* and *W_P_* were the weight of **M** and **P**, respectively. In these equations, *m_i_*, *C(i)_M_* and *C(i)_P_* were determined as in Section 2.3.4. *Y_i_* could be calculated according to Equation (1). Equation (2) transformed into Equation (3). *W_M_* and *W_P_* were calculated using Equations (3) and (4).

Based on each monoterpene glycoside, *Y_i_* could be obtained, and according to the equations, the *W_M_* and *W_P_* in GZFLW was calculated. Taking **1** as an example, the results are shown in Table 5. The accuracy was described by related error (RE), all within 10%. When the weight of **M** and **P** in GZFLW were calculated through all four monoterpeneglycosides, the results would be more available and effective. The results of the method validation indicated that the method developed above could be successfully applied for the analysis of the weight of **M** and **P** in GZFLW.

**Table 5:** The result of analysis of the weight of **M** and **P** in GZFLW (g).

Sample		NO.1	NO.2	NO.3	NO.4	NO.5	NO.6	NO.7	NO.8	NO.9	NO.10	NO.11
**M**	Act.	0.5021	0.5572	0.6734	0.7481	0.8807	1.0151	1.1223	1.2482	1.3371	1.4213	1.5046
	Cal.	0.4617	0.5113	0.6690	0.7038	0.9352	1.0056	1.0570	1.2370	1.2983	1.4639	1.5237
	RE%	8.05	8.24	0.65	5.92	6.19	0.94	5.82	0.89	2.90	3.00	1.27
**P**	Act.	1.4996	1.4334	1.3308	1.2468	1.1251	1.0053	0.8956	0.7448	0.6712	0.5658	0.5042
	Cal.	1.6114	1.5061	1.2983	1.3671	1.0491	1.0321	0.9307	0.7724	0.7317	0.5164	0.4567
	RE%	7.46	5.07	2.44	9.65	6.76	2.67	3.92	3.70	9.01	8.73	9.42

Cal: calculated value, Act: actual value, RE: related error.

## 3. Experimental Section

### 3.1. Materials and Reagents

The reference standard of **1** was isolated from **P** in our laboratory, while **3** and **4** were isolated from **M**. The structures were determined by spectral methods, including MS, 1H-NMR and 13C-NMR. The data were consistent with those reported in the literature [26,27,28]. The purity of the reference standard was found to be above 98%, based on a peak area normalization method using HPLC-DAD and HPLC-ESI-TOF-MS. The extractions and isolations were as follows:

The air-dried **P** (500 g) was extracted with EtOH-H_2_O (70:30, 3 × 5 L) under refluxconditions for 3 h. The combined EtOH extracts were concentrated *in vacuo* to generate a crude residue (82.7 g) that was suspended in H_2_O (200 mL). The suspension was extracted with *n*-BuOH-EtOAc (4:1, 5 × 1 L). The combined *n*-BuOH-EtOAc portion (41.8 g) was separated by a silica gel CC eluted with CH_2_Cl_2_-MeOH (from 100:0 to 0:100) to yield 24 fractions (A-X). Fraction N (0.3 g) was separated over preparative RP-HPLC (MeCN-H_2_O, 44:56, *v*/*v*) to afford **1** (34.2 mg).

Crushed air-dried Moutan cortex (500 g) was extracted with EtOH-H_2_O (70:30, 3 × 5 L) under reflux conditions for 3 h. The combined EtOH extracts were concentrated *in vacuo* to generate a crude residue (43.6 g) that was suspended in H_2_O (150 mL). The suspension was extracted with *n*-BuOH-EtOAc (4:1, 5 × 1 L). The combined *n*-BuOH-EtOAc portion (20.5 g) was subjected to silica gel column chromatography with a gradients of CH_2_Cl_2_-MeOH (from 100:0 to 0:100) to afford 22 fractions (F_1_–F_22_). Fraction 14 (0.3 g) was separated by preparative RP-HPLC, with MeCN/H_2_O (40:60, *v*/*v*) as mobile phase, to afford **3** (27.6 mg). Fraction 10 (0.2 g) was separated via preparative RP-HPLC (MeCN-H_2_O, 40:60, *v*/*v*) to obtain **3** (24.8 mg).

The reference standard of **2** was purchased from the Chinese National Institute for Control of Pharmaceutical and Biological Products (Beijing, China). Cinnamomiramulus, Poriacocos, Moutan cortex, Paeoniae radix alba, and Persicae semen were provided by Jiangsu KanionPharmaceut Co. Ltd. (Lianyungang, Jiangsu, China). The five crude dried parent plants were pulverized and sifted through 24 mesh sieve before analysis. HPLC grade methanol was obtained from Mallinckrodt Baker Inc. (Phillipsburg, PA, USA). Phosphoric acid (HPLC grade) was purchased from Tianjin Kemiou Chemical Reagent Co. Ltd. (Tianjin, China). HPLC grade water was prepared with redistilled water equipment (Shanghai, China) in this study. Acetone and other solvents of analytical grade were obtained from Tianjin Fuyu Chemical Reagent Co. Ltd. (Tianjin, China).

### 3.2. Sample Preparation

#### 3.2.1. Preparation of GZFLW (Series Ratio of **M** and **P**) and Negative Control

GZFLW and the negative control (without **M** and **P**) were prepared in accordance with the process stated in the Chinese Pharmacopeia (2010 Edition) [1]. The negative control was used to prove that the otherthree herbs didn’t contain theanalyzed compoundsgalloylpaeoniflorin (**1**), paeoniflorin (**2**), mudanpioside C (**3**) and benzoylpaeoniflorin (**4**).

#### 3.2.2. Preparation of the Sample and Negative Control Solutions

The accurately weighed GZFLW (5 g), negative control (3 g), **M** (1 g) and **P** (1 g) were extracted by methanol for 3 h in 60 mL soxhlet flask, respectively. After cooling, the solutions were removed and diluted with methanol to 100 mL in a volumetric flask, and then filtered through a 0.22 μm Millipore filter.

#### 3.2.3. Preparation of the Standard Solutions

Stock solutions of **1**, **2**, **3** and **4** were prepared by dissolving the appropriate amount of each standard compound in methanol. A mixed working standard solution was prepared by diluting a mixture of each reference compound stock solution with methanol. The concentrations of the mixed working standard solutions were 22.05 μg/mL for **1**, 332.75 μg/mL for **2**, 5.95 μg/mL for **3** and 45.6 μg/mL for **4**, respectively. These solutions were stored in a refrigerator at −20 °C and brought to room temperature before analysis.

#### 3.2.4 Apparatus and Chromatographic Conditions

Waters-2690 Alliance HPLC instrument (Waters Corporation, Milford, MA, USA) was used in this study, equipped with an online degasser, an auto injector, a column heater and a 2996 photodiode array detector (DAD). UV-Visible detection was achieved over the wavelength range of 200–600 nm. The detection wavelength was 270 nm for **1** and **2**, while **3** and **4** were monitored at 254 nm and 230 nm, respectively. Chromatographic separation was performed at 30 °C on a SunFire^TM^ C18 reverse phase column (5.0 μm, 150 mm×4.6 mm I.D.), with (A) methanol and (B) 0.3% phosphoric acid as the mobile phase. Gradient programming was performed with linear gradient (5%–8% A at 0–8 min, 8%–24% A at 8–13 min, 24%–29% A at 13–30 min, 29%–42% A at 30–35 min, 42%–51% A at 35–60 min). The flow rate was 1.0 mL/min, and the injection volume was 10 μL. Therefore, the monoterpene glycosides were well separated in the above chromatographic conditions.

## 4. Conclusions

In our study, a new and reliable analytic method was developed and validated to detect the weight of **M** and **P** in GZFLW by HPLC-DAD through the analysis of the content of **1**, **2**, **3** and **4** in **M**, **P** and GZFLW with different ratios of **M** and **P**. The method was simple, but has been demonstrated to be of excellent precision and accuracy. We successfully used the method by measuring the weight of **M** and **P** in GZFLW. All relative errors (RE) of the weight of **M** and **P** in GZFLW were within 10%. The result has shown that the method could besuccessfully applied for the content analysis of **M** and **P** inGZFLW. Moreover, it may be widely used to control the quality of proprietary Chinese medicines containing herbs of the same genus or family in industrial production.
